# The state of the science on the health benefits of blueberries: a perspective

**DOI:** 10.3389/fnut.2024.1415737

**Published:** 2024-06-11

**Authors:** April J. Stull, Aedín Cassidy, Luc Djousse, Sarah A. Johnson, Robert Krikorian, Johanna W. Lampe, Kenneth J. Mukamal, David C. Nieman, Kathryn N. Porter Starr, Heather Rasmussen, Eric B. Rimm, Kim S. Stote, Christy Tangney

**Affiliations:** ^1^Department of Human Sciences and Design, Baylor University, Waco, TX, United States; ^2^Institute for Global Food Security, Queen's University Belfast, Belfast, United Kingdom; ^3^Department of Medicine at Brigham and Women’s Hospital and Harvard Medical School, Boston, MA, United States; ^4^Department of Food Science and Human Nutrition, Colorado State University, Fort Collins, CO, United States; ^5^Department of Psychiatry & Behavioral Neuroscience, University of Cincinnati Academic Health Center, Cincinnati, OH, United States; ^6^Public Health Sciences Division, Fred Hutchinson Cancer Center, Seattle, WA, United States; ^7^Beth Israel Deaconess Medical Center, Harvard Medical School, Boston, MA, United States; ^8^Human Performance Laboratory, North Carolina Research Campus, Appalachian State University, Kannapolis, NC, United States; ^9^Department of Medicine, Duke University School of Medicine and Geriatric, Research, Education and Clinical Center, Durham VA Health Care System, Durham, NC, United States; ^10^Department of Nutrition and Health Sciences, University of Nebraska-Lincoln, Lincoln, NE, United States; ^11^Departments of Epidemiology & Nutrition, Harvard T.H. Chan School of Public Health, Channing Division of Network Medicine, Brigham and Women's Hospital and Harvard Medical School, Boston, MA, United States; ^12^Albany Stratton VA Medical Center, Albany, NY, United States; ^13^Department of Clinical Nutrition, Rush University, Chicago, IL, United States

**Keywords:** blueberry, anthocyanins, cardiovascular disease, cognitive function, exercise, gut microbiome, diabetes, vascular function

## Abstract

Mounting evidence indicates that blueberry consumption is associated with a variety of health benefits. It has been suggested that regular consumption of blueberries can support and/or protect against cardiovascular disease and function, pre-diabetes and type 2 diabetes, and brain and cognitive function in individuals with health conditions and age-related decline. Further, mechanistic investigations highlight the role of blueberry anthocyanins in mediating these health benefits, in part through interactions with gut microbiota. Also, nutritional interventions with blueberries have demonstrated the ability to improve recovery following exercise-induced muscle damage, attributable to anti-inflammatory effects. Despite these advancements in blueberry health research, research gaps persist which affects the generalizability of findings from clinical trials. To evaluate the current state of knowledge and research gaps, a blueberry health roundtable with scientific experts convened in Washington, DC (December 6–7, 2022). Discussions centered around five research domains: cardiovascular health, pre-diabetes and diabetes, brain health and cognitive function, gut health, and exercise recovery. This article synthesizes the outcomes of a blueberry research roundtable discussion among researchers in these domains, offering insights into the health benefits of blueberries and delineating research gaps and future research directions.

## 1 Introduction

Blueberries are a rich source of vitamins, minerals, dietary fiber, and polyphenols (1). Their high content of polyphenols, in particular anthocyanins, can play a role in promoting human health and reducing chronic disease risk. Habitual intake of anthocyanins has been associated with a range of potential health benefits including but not limited to reduced risk of overall mortality, cardiovascular disease (CVD) and related events, type 2 diabetes, and improved cognitive function (2–8). Additionally, there have been improvements in cardiovascular and metabolic risk factors such as blood concentrations of total cholesterol, lipoproteins, and inflammatory biomarkers, as well as improved vasodilation, blood flow, and elasticity of blood vessels (2, 8, 9). Anthocyanins undergo extensive metabolism after ingestion, leading to the production of gut microbial- and phase II metabolism-derived compounds that may play a key role in the observed health benefits (10, 11). The interactions between polyphenols, including anthocyanins, gut microbiota, and their metabolites is an emerging area of research (11). Moreover, evolving research on the role of anthocyanins in exercise recovery suggests their role as a countermeasure to exercise-induced inflammation (12).

**Figure 1:**
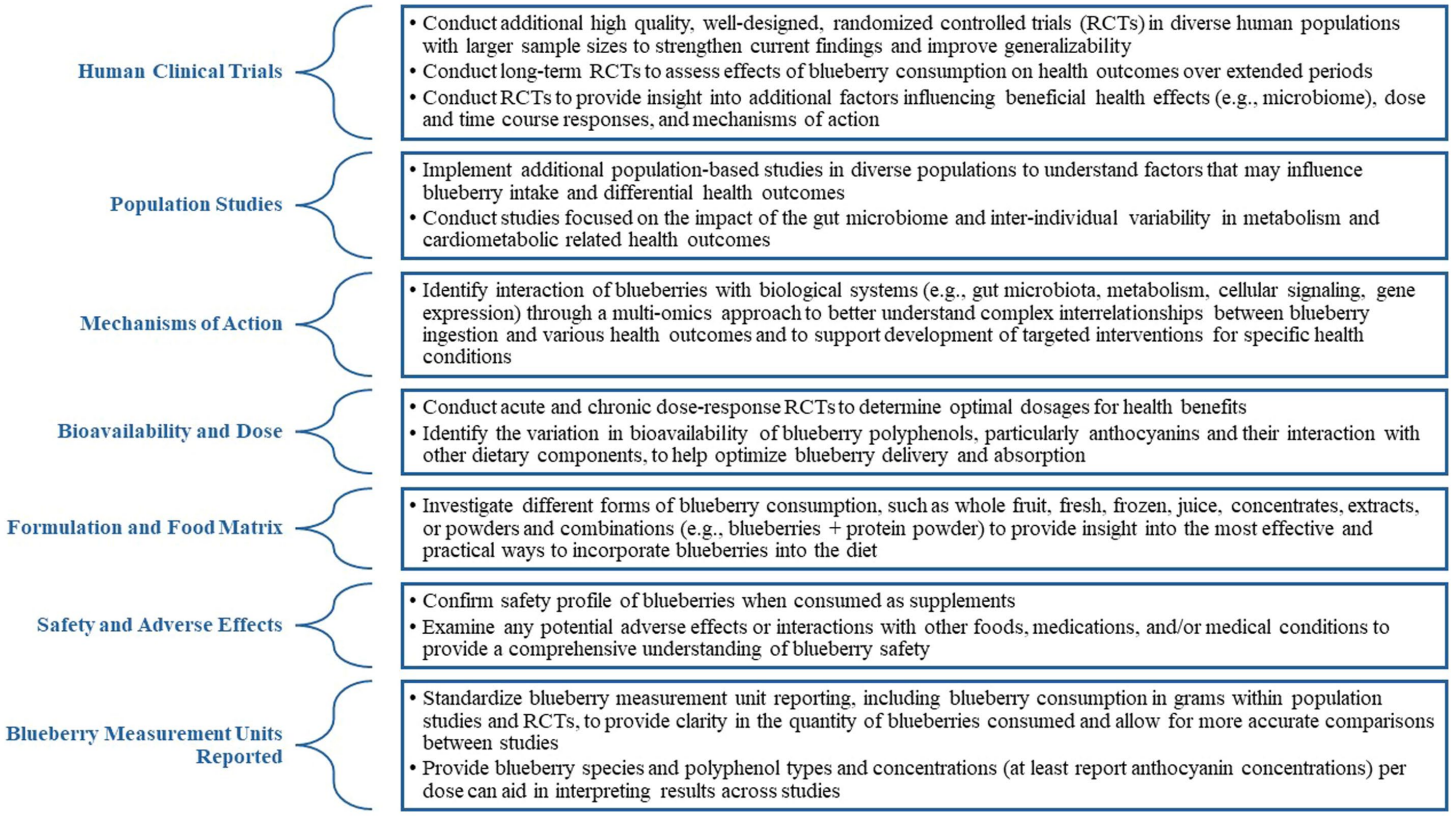
Recommendations for additional research on blueberry consumption and health benefits.

A roundtable of scientific experts convened in Washington, DC in December 6–7, 2022 to discuss the science related to the health benefits of blueberries. These experts represented the fields of nutrition, dietetics, food science, nutritional biochemistry and metabolism, exercise science, cardiovascular health and physiology, cognitive function, clinical and translational sciences, epidemiology, and public health. The following research domains were explored: cardiovascular health, pre-diabetes and diabetes, brain health and cognitive function, gut health, and exercise recovery. The purpose of the roundtable discussion was to evaluate the current state of the science on the health benefits of blueberries and address the research gaps and future directions for each identified health benefit. This article summarizes a roundtable discussions among scientific experts, providing insights into the health benefits of blueberries while outlining the research gaps and suggesting future research directions within each research domain. Additionally, the roundtable discussion encompassed perspectives on adding blueberries to the United States Dietary Guidelines and establishing blueberry consensus statements. A summary of recommendations for further research on the health benefits of blueberries is presented in Figure 1.

## 2 Health benefits of blueberries

### 2.1 Cardiovascular health

The predominant focus of blueberry research has been on cardiovascular health, mainly from long-term prospective cohort studies of CVD and shorter-term experimental studies evaluating effects on cardiometabolic markers. In the Nurses’ Health Study II (NHS), consumption of >3 servings per week of strawberries and blueberries compared with ≤1 time per week was associated with a trend towards a 34% lower risk of myocardial infarction (13). Higher habitual intakes of anthocyanins were associated with a 32% reduction in risk of myocardial infarction when participants in the highest and lowest quintiles were compared. In the combined NHS and Health Professionals Follow-up Study, the consumption of >1 serving of blueberries per week was associated with a 10% lower risk in hypertension when compared with no blueberry intake (14). Additionally, higher habitual anthocyanin intakes were associated with an 8% reduction in risk of hypertension (comparing intakes in quintile 5 vs. quintile 1).

Several meta-analyses have shown beneficial effects of blueberries on cardiometabolic markers. Xu et al. (8) conducted a meta-analysis of 44 randomized controlled trials (RCTs) and 15 prospective cohort studies and found that consuming anthocyanin-rich berries (i.e., blueberries, cranberries, bilberries, and blackcurrant) was associated with lower blood total cholesterol and the pro-inflammatory marker C-reactive protein. Also, high intake of dietary anthocyanins was associated with lower coronary heart disease (CHD) risk and total CVD incidence and mortality (8). A previous meta-analysis by Carvalho et al. (15) included 18 blueberry-intervention RCTs and they found that blueberries significantly decreased blood lipids (i.e., total cholesterol and LDL cholesterol) and diastolic blood pressure. Martini et al. (9) conducted a systematic review encompassing 45 intervention studies and the consumption of blueberries had a positive impact on vascular function with mixed results for oxidative stress, inflammation, and blood pressure. Moreover, Stote et al. (16) conducted a systematic review evaluating berries (including blueberries) and blood and urine biomarkers of oxidative stress (e.g., antioxidants, DNA damage, isoprostanes, malondialdehyde and oxidized LDL). A total of 56 different biomarkers of oxidative stress were evaluated and many were beneficially impacted by experimentally increasing berry intake. It is important to note that studies that did not observe a significant impact evaluated oxidative stress as a secondary outcome measurement and the studies were not statistically powered to evaluate this outcome.

RCTs have shown that consuming blueberries daily for up to 24 weeks can have beneficial effects on cardiometabolic markers. The blueberry doses ranged from the equivalent of 1–2 cups or 150–350 g fresh blueberries which provided anthocyanin contents ranging from 224–742 mg. Several RCTs have suggested that regular consumption of blueberries may help lower blood pressure in individuals who are sedentary (17, 18), have metabolic syndrome (19), and/or are postmenopausal women with above-normal blood pressure (i.e., elevated blood pressure or hypertension) (20). However, other studies reported no effect of blueberry intake on blood pressure (21–23). Blueberries have been shown in acute and chronic intervention RCTs to improve vascular function, including endothelial function (21–25) and arterial stiffness (17, 20, 21). One study in postmenopausal women with elevated blood pressure or stage 1-hypertension showed that improvements in endothelial function were mediated directly through reductions in oxidative stress (23). Acute blueberry intake in varying doses, ranging from the equivalent of 100–240 g fresh blueberries (providing 129–310 mg anthocyanins), exhibited a dose-dependent improvement in vascular function among healthy men (26). Furthermore, studies strongly suggest that anthocyanins and their metabolites are key mediators of blueberries’ vascular-protective effects (27, 28). In a particular study, a low dose of 160 mg purified anthocyanins acutely improved endothelial function in healthy young men to the same extent as the equivalent of 0.5 cups of fresh blueberries containing 150 mg anthocyanins (28). However, an equivalent dose of 0.5 cups of fresh blueberries providing 182 mg anthocyanins did not improve endothelial function and other cardiometabolic biomarkers in middle-aged/older adults with metabolic syndrome (21). Thus, higher doses may be required to achieve health benefits in aging individuals with cardiometabolic risk factors than in healthy adults.

#### 2.1.1 Research gaps and future directions

A higher (≥ 1–3 servings/week) habitual blueberry intake is associated with cardiovascular health benefits based on population studies. Findings from acute and chronic blueberry consumption studies are promising for CVD risk and health, particularly with regard to vascular function. More research is warranted for blood pressure, CVD and cardiovascular risk and vascular function related to oxidative stress, inflammation, antioxidant defense, and nitric oxide production/bioavailability in humans. Additional research is particularly needed to investigate the longer-term impact of daily blueberry intake (i.e., > 6 months) on CVD risk and vascular function and to understand individual factors associated with beneficial health outcomes. Importantly, RCTs evaluating cardiovascular health benefits of blueberries have been heterogeneous with respect to study population, methodology, interventions, among other factors and could be a factor contributing to differential outcomes in cardiovascular health among studies. Inclusion of repeated comprehensive measurements of cardiovascular health and function are needed (e.g., clinic, home and/or 24-h ambulatory blood pressure, biological sample markers, endothelial function, and arterial stiffness), particularly in studies assessing outcomes with inconsistent efficacy (e.g., blood pressure). Population and community-based studies that focus on blueberries (not a mixture of berries) and cardiovascular health are warranted. Also, studies are limited that directly evaluate the effects of blueberry intake on vascular oxidative stress, inflammation, antioxidant defense, and nitric oxide production/bioavailability independently and as possible mechanisms for improvements in vascular function in diverse populations that have prevalent hypertension and/or CVD.

### 2.2 Pre-diabetes and type 2 diabetes

In epidemiological studies, a higher habitual intake of blueberries is associated with a lower risk of type 2 diabetes (7, 29, 30). Specify, in a prospective longitudinal cohort study, ≥ 2 blueberry servings/week was associated with a lower risk of developing type 2 diabetes when compared to infrequently or not consuming blueberries (< 1 serving / month) (7). Moreover, RCTs have investigated the effects of daily blueberry consumption on glucose regulation and insulin resistance over periods of 6 to 24 weeks. Blueberry doses ranged from the equivalent of 0.5–2 cups or 75–300 g fresh blueberries, providing anthocyanin contents ranging from 182 mg to 668 mg. In an RCT with adults who had obesity and insulin resistance (pre-diabetes), insulin sensitivity was assessed by hyperinsulinemic-euglycemic clamps and improved after 6 weeks of daily blueberry intake (equivalent of 300 g fresh blueberries; providing 668 mg anthocyanins) (31). Contrarily, other studies observed no discernible differences in insulin sensitivity in periods of 6 weeks and 24 weeks among participants with pre-diabetes (equivalent of 300 g and 150 g fresh blueberries, respectively; containing 580 mg and 364 mg anthocyanins, respectively) (21, 22). Insulin sensitivity was measured by frequently sampled intravenous glucose tolerance test (FSIVGTT), homeostasis model assessment of insulin resistance (HOMA-IR), and hyperinsulinemic euglycemic clamp in a subset of participants. Blood hemoglobin A1c and fructosamine were reduced after consuming blueberries daily for 8 weeks in men with type 2 diabetes (32) whereas other studies in men and women with pre-diabetes observed no changes in HbA1c (21, 33). Most blueberry intervention studies have not observed changes in fasting glycemia and insulinemia (21, 22, 31, 32). However, a 12-week blueberry intervention study demonstrated lower fasting blood insulin levels in a population with pre-diabetes and subjective cognitive decline while no additional changes in fasting blood glucose levels and HOMA-IR were noted (33). Also, an acute study found consuming blueberries (equivalent to 1 cup or 150 g fresh blueberries; containing 364 mg anthocyanins) reduced postprandial blood glucose and insulin concentrations over 24 h after consuming an energy-dense high-fat/high-sugar meal (34).

#### 2.2.1 Research gaps and future directions

A higher (≥ 2 servings/week) habitual blueberry intake is associated with a lower risk of type 2 diabetes based on population studies. However, the current body of evidence on the impact of blueberry consumption on glucose regulation and insulin resistance in adults with dysglycemia yields mixed results, making it challenging to draw a definitive conclusion from the RCTs. Further research is required to explore the impact of blueberries on glucose intolerance and/or insulin resistance, with a particular focus on extended durations (> 6 months) and understanding individual factors associated with beneficial outcomes. It is possible that participants with normal or near normal blood glucose levels may experience only marginal or negligible improvements in glycemia after consumption of blueberries. Therefore, if individuals with pre-diabetes are included in future RCTs, it is recommended to recruit individuals based on a higher fasting blood glucose level (110–125 mg/dL) that is closer to the mid to high end range for pre-diabetes. Furthermore, blood HbA1c levels in the prediabetes range (5.7–6.4%) have not been consistently assessed in studies, despite their greater stability than fasting glucose levels, and should be included in future studies as an important measurement of long-term glycemic changes. Additionally, because blueberries contain calories and carbohydrates, future studies evaluating their impact on prediabetes and diabetes should consider reducing carbohydrates within the diet to ensure a balance between energy intake and carbohydrate consumption. By implementing these recommendations, researchers can gather more comprehensive and reliable data on the potential long-term benefits of blueberries in relation to glucose regulation and insulin resistance in individuals with dysglycemia and the possibility of delaying the onset of type 2 diabetes.

### 2.3 Brain health and cognitive function

There is evidence from population studies that higher berry (i.e., blueberries and strawberries) intake has been associated with a slower rate of cognitive decline (≥2 servings of strawberries/week and ≥1 serving of blueberries/week) (35), higher probability of healthy aging with the absence of major chronic diseases and physical and cognitive disability (≥ 2 servings of berries/week) (36), and lower risk of Parkinson’s disease, a progressive neurodegenerative disorder (2–4 servings of berries/week) (37). Several reviews have concluded that blueberries exhibit small to moderate beneficial effects on cognitive function in the aging population (2, 3, 6).

In aged mice and rats, blueberry intake improved cognitive performance and corrected deficits and/or age-related cognitive decline (38–42). Also, in pre-clinical studies, blueberry intake protected against cognitive deficits associated with brain insults (43–45) and poor diet (i.e., high linoleic acid intake) (46). Clinical studies have demonstrated positive effects of daily freeze-dried blueberry consumption with various doses, ranging from 0.5–1 cup or 75–180 g fresh blueberry equivalent (providing 140–461 mg anthocyanins), on cognitive performance over intervention durations from 12 to 24 weeks. One study observed similar benefits with 444–621 mL/day of blueberry juice (604–845 g fresh blueberry equivalent; providing 428–598 mg anthocyanins) (47). These effects have been observed in middle-aged and older adults who were cognitively unimpaired (i.e., nonpathological aging) (18, 33, 48, 49) as well as in older, cognitively impaired individuals (i.e., pathological aging) (47, 50, 51). Mechanisms associated with the cognitive benefits of blueberry consumption might include modulation of metabolic disturbances such as reducing hyperinsulinemia (33), enhancing cerebral activation and blood flow (52, 53), improving vascular function (18), and reducing inflammation and oxidative stress (43, 54).

#### 2.3.1 Research gaps and future directions

A higher (≥ 1–2 servings/week) habitual blueberry intake is associated with brain health and cognitive function benefits based on population studies. Blueberry consumption can enhance aspects of cognitive performance in the contexts of aging and cognitive impairment resulting from brain injury or metabolic and vascular disturbances. However, further investigation is needed to elucidate the underlying mechanisms and pathways involved in enhanced cognitive performance. Useful approaches might include relating functional brain imaging measures and other biomarkers with cognitive performance and anthocyanin levels.

## 3 Emerging research

### 3.1 Gut health

While there is no consensus definition for gut health, it is typically defined as absence of gastrointestinal (GI) symptoms (e.g., abdominal pain, diarrhea), disease (e.g., inflammatory bowel disease, CVD, diabetes, colon cancer), and unfavorable GI conditions (e.g., increased permeability, mucosal inflammation) (55, 56). Functional gastrointestinal disorders (FGID), including irritable bowel syndrome, functional dyspepsia, and functional constipation, are common complaints in GI clinics (57). To date, few human studies have examined the effect of blueberry intake on FGID outcomes or GI symptoms. An RCT crossover study showed that consuming blueberries led to improvements in abdominal symptom relief and quality of life scores compared to a placebo among individuals with FGID, with no effect on fructose fermentation or stool consistency (58).

Improving health through modulation of the gut microbiota is a rapidly developing research area for many chronic diseases, and the gut is a potential site of action for ingested blueberries. The gut microbiota can be altered favorably in response to certain dietary changes, including chronic consumption of blueberries. Blueberry anthocyanins and other polyphenols undergo extensive metabolism by the intestinal microbiota, raising the possibility that polyphenol metabolites induced by microbiota may mediate the health-promoting effects of blueberries (59). In a systematic review of 16 animal studies, blueberry consumption improved gut health by improving intestinal morphology, reducing gut permeability, suppressing oxidative stress, ameliorating gut inflammation, and modulating the composition and function of gut microbes (60). Experimental studies in *in vitro* and animal models suggest that intake of blueberry extract may positively influence gut epithelial function and barrier integrity (61, 62). In rodents, changes in gut microbiota composition were modulated by blueberry intake (63–65) and the consumption of blueberries effectively suppressed oxidative damage and inflammation in the colon in mice with induced ulcerative colitis (66).

Limited intervention RCTs in humans have demonstrated that consuming blueberries for 6 weeks resulted in significant, but often modest and variable changes in gut microbiota composition (250 g fresh blueberry equivalent) (67) and bifidobacteria specifically (150 g fresh blueberry equivalent; providing 375 mg anthocyanins) (68). Also, one study found no major changes in gut microbiota composition after consuming a fresh blueberry equivalent dose of 180 g (providing 302 mg anthocyanins) for 12 weeks (18). However, increases in beneficial bacteria (e.g., *Ruminiclostridium* and *Christensenellaceae*) were observed as well as correlations between several butyrate-producing bacteria and measures of endothelial and cognitive function.

Gut microbial dysbiosis can lead to cardiometabolic disorders, and evidence suggests chronic blueberry consumption may confer favorable health effects and reduce chronic disease risk through interactions with the gut microbiome. Animal models suggest that blueberries affect downstream markers of health in part through gut microbial mechanisms, but more research is needed to confirm these effects in humans. These gut microbial changes with blueberries have been associated with reduced systemic inflammation (59, 61), body weight and adipose tissue (69–72), oxidative stress (72), and liver injury (liver fibrosis) (73). Also, gut microbiota changes were associated with improved insulin sensitivity / glucose tolerance (61, 71, 74).

#### 3.1.1 Research gaps and future directions

Chronic blueberry consumption can modulate the gut microbiota. However, more intervention RCTs in humans that characterize the effects of blueberry intake on indicators of gut health are warranted. Also, dose-dependent effects of blueberries and their components (e.g., polyphenols, fiber, and sugars) on gut microbiota need to be evaluated to determine the optimal amount of blueberries necessary to elicit beneficial effects. Further, the interactions between blueberries and the gut microbiota/microbiome and resultant effects in improving human health still need to be elucidated. Understanding how blueberries influence the composition, diversity, and metabolic functions of the gut microbiota can provide insights into the underlying mechanisms and pathways by which blueberries exert their potential health benefits.

### 3.2 Exercise recovery

Most studies involving exercise, and blueberry or anthocyanin intake have focused on physiological stress (e.g., inflammation and oxidative stress) outcomes. Multiple literature reviews have concluded that increased intake of blueberries, anthocyanins, and polyphenols may have a small, variable, or null effect on attenuating exercise-induced muscle soreness, damage, and dysfunction (12, 75–82).

Blueberry intake has been associated with reduced blood inflammatory (83) and oxidative stress (84–86) biomarkers, and an increase in blood anti-inflammatory cytokines (84), especially after metabolically demanding exercise bouts. An RCT showed that ingestion of 1 cup or 150 g fresh blueberry equivalent per day (providing 345 mg anthocyanins) for 2-weeks increased plasma levels of gut-derived phenolics and countered post-exercise increases in plasma levels of 10 proinflammatory lipid mediators (oxylipins) following a 75-km cycling bout (87). Similarly, another RCT showed that consuming an equivalent of 1 cup or 150 g fresh blueberries per day (providing 280 mg anthocyanins) during an 18-day period improved resolution of inflammation by lowering pro-inflammatory and increasing anti-inflammatory lipid mediators during the 4-day recovery period after an acute 90-min bout of unaccustomed exercise in untrained adults (88). However, one study did not observe any reductions in exercise-induced oxidative stress and/or inflammation following 8 weeks of blueberry consumption (equivalent to 2 cups or 300 g fresh blueberries) in untrained individuals (89). Overall, blueberries may be beneficial in mitigating inflammation and improving recovery following metabolically demanding exercise bouts.

#### 3.2.1 Research gaps and future directions

Blueberry consumption may improve inflammation resolution after metabolically demanding exercise bouts. However, further investigation on the influence of blueberry ingestion on exercise-induced inflammation and physiological stress is needed because previous studies varied widely in research designs, dosing strategies, and quality of outcome measurements. Also, further investigation of studies with longer blueberry intake periods (>2 weeks) and the incorporation of metabolically challenging exercise bouts are warranted.

## 4 Exploring dietary guidelines and consensus statements for blueberries

It remains important to continually amass a comprehensive body of evidence regarding the human health benefits of blueberries to guide future dietary recommendations in the United States. Currently, the 2020–2025 Dietary Guidelines for Americans recommend that adults consume approximately 1.5–2 cup equivalents per day of whole fruits and 100% fruit juice (90). However, dietary guidance should extend beyond general fruit recommendations and incorporate specific fruit subgroup categories based on type (e.g., berries, citrus fruits, tropical fruits, melons, etc.) or color (e.g., blue/purple, red, yellow, etc.) as they currently do for vegetables. Encouraging the regular inclusion of fruits from various categories (e.g., type or color) ensures a diverse and nutritionally rich intake. Different types and colors of fruits offer varying profiles of nutrients and health-promoting phytochemicals such as polyphenols (91). The recently published Nordic Nutrition Recommendations underscores the significance of including berries in the diet for health benefits, and they recommend a daily intake of 500–800 g or more of a variety of vegetables, fruits, and berries (92).

It is important to note that whole fruit (e.g., fresh, frozen, and freeze-dried) and 100% fruit juices contain numerous nutrients and phytochemicals which may have additive and/or synergetic effects that may not occur with administration of isolated compounds like anthocyanins. Notably, most clinical trials with blueberry interventions evaluated in this perspective utilized the whole fruit in the form of freeze-dried powder. The potential health benefits of dietary supplements containing anthocyanins extracted from blueberries is not well-established. There could be safety concerns associated with purified anthocyanin supplementation in humans, although this has not been investigated. While dietary supplements may offer convenience and targeted phytochemical delivery, they are not necessarily a substitute for whole fruits or a balanced diet. If fresh blueberry consumption is limited due to their seasonal availability and moderate shelf-life, individuals can also consume frozen or freeze-dried blueberries including blueberry powder (93, 94).

While research highlights the positive impact of blueberries on various aspects of health, there is currently no consensus recommendation for the specific quantity of blueberries to incorporate into an individual’s diet. Further, it may be that recommendations will need to be tailored to specific disease and health states or to different age or sex groups. As our understanding of the health benefits of blueberries advances, it may be possible to reach a consensus on the recommended daily or weekly intake. Establishing an optimal amount may not only enhance public awareness, but also aid individuals in making informed decisions for better health outcomes. More well-designed RCTs are needed to strengthen the evidence base for systemic reviews and meta-analyses. These are important components in the development of dietary recommendations, clinical guidelines, and consensus statements. Additionally, understanding the health impacts of blueberries at different doses (alone and in combination with other foods), phytochemicals/bioactive compounds, within the context of food products and dietary patterns, and across various populations (e.g., health/disease states, sex/gender, race/ethnicity, etc) can help inform recommendations for blueberry consumption based on precision and personalized nutrition approaches.

## 5 Conclusion

In summary, promising evidence suggests that blueberry intake can be beneficial with respect to cardiovascular health, pre-diabetes and type 2 diabetes, brain health and cognitive function, gut health, and exercise recovery. In addition, the blueberry health benefits research roundtable discussion highlighted research gaps and provided recommendations that can help guide researchers and funding agencies. Addressing the identified research gaps will not only advance our understanding of the health benefits linked with blueberries but also provide evidence supporting their inclusion in national dietary guidelines and clinical practice guidelines, ultimately leading to improved public health outcomes.

## Data availability statement

The original contributions presented in the study are included in the article/supplementary material, further inquiries can be directed to the corresponding author.

## Author contributions

AS: Conceptualization, Writing – original draft. AC: Writing – review & editing. LD: Writing – review & editing. SJ: Writing – review & editing. RK: Writing – review & editing. JL: Writing – review & editing. KM: Writing – review & editing. DN: Writing – review & editing. KP: Writing – review & editing. HR: Writing – review & editing. ER: Writing – review & editing. KS: Writing – review & editing. CT: Writing – review & editing.

## References

[1] Golovinskaia O Wang CK. Review of functional and pharmacological activities of berries. Molecules. (2021) 26:3904. doi: 10.3390/molecules26133904, PMID: 34202412 PMC8271923

[2] Ahles S Joris PJ Plat J. Effects of berry anthocyanins on cognitive performance, vascular function and Cardiometabolic risk markers: a systematic review of randomized placebo-controlled intervention studies in humans. Int J Mol Sci. (2021) 22:6482. doi: 10.3390/ijms22126482, PMID: 34204250 PMC8234025

[3] Bell L Williams CM. Blueberry benefits to cognitive function across the lifespan. Int J Food Sci Nutr. (2021) 72:650–2. doi: 10.1080/09637486.2020.1852192 33249925

[4] Bondonno NP Liu YL Zheng Y Ivey K Willett WC Stampfer MJ. Change in habitual intakes of flavonoid-rich foods and mortality in US males and females. BMC Med. (2023) 21:181. doi: 10.1186/s12916-023-02873-z, PMID: 37173745 PMC10182674

[5] Grosso G Micek A Godos J Pajak A Sciacca S Galvano F. Dietary flavonoid and Lignan intake and mortality in prospective cohort studies: systematic review and dose-response Meta-analysis. Am J Epidemiol. (2017) 185:1304–16. doi: 10.1093/aje/kww207, PMID: 28472215

[6] Hein S Whyte AR Wood E Rodriguez-Mateos A Williams CM. Systematic review of the effects of blueberry on cognitive performance as we age. J Gerontol A Biol Sci Med Sci. (2019) 74:984–95. doi: 10.1093/gerona/glz082, PMID: 30941401

[7] Wedick NM Pan A Cassidy A Rimm EB Sampson L Rosner B. Dietary flavonoid intakes and risk of type 2 diabetes in US men and women. Am J Clin Nutr. (2012) 95:925–33. doi: 10.3945/ajcn.111.028894, PMID: 22357723 PMC3302366

[8] Xu L Tian Z Chen H Zhao Y Yang Y. Anthocyanins, anthocyanin-rich berries, and cardiovascular risks: systematic review and Meta-analysis of 44 randomized controlled trials and 15 prospective cohort studies. Front Nutr. (2021) 8:747884. doi: 10.3389/fnut.2021.747884, PMID: 34977111 PMC8714924

[9] Martini D Marino M Venturi S Tucci M Klimis-Zacas D Riso P. Blueberries and their bioactives in the modulation of oxidative stress, inflammation and cardio/vascular function markers: a systematic review of human intervention studies. J Nutr Biochem. (2023) 111:109154. doi: 10.1016/j.jnutbio.2022.109154, PMID: 36150681

[10] Cassidy A Minihane AM. The role of metabolism (and the microbiome) in defining the clinical efficacy of dietary flavonoids. Am J Clin Nutr. (2017) 105:10–22. doi: 10.3945/ajcn.116.136051, PMID: 27881391 PMC5183723

[11] Woolf EK Lee SY Ghanem N Vazquez AR Johnson SA. Protective effects of blueberries on vascular function: a narrative review of preclinical and clinical evidence. Nutr Res. (2023) 120:20–57. doi: 10.1016/j.nutres.2023.09.007, PMID: 37913730 PMC12046616

[12] Gonçalves AC Gaspar D Flores-Félix JD Falcão A Alves G Silva LR. Effects of functional Phenolics dietary supplementation on Athletes' performance and recovery: a review. Int J Mol Sci. (2022) 23:4652. doi: 10.3390/ijms23094652, PMID: 35563043 PMC9102074

[13] Cassidy A Mukamal KJ Liu L Franz M Eliassen AH Rimm EB. High anthocyanin intake is associated with a reduced risk of myocardial infarction in young and middle-aged women. Circulation. (2013) 127:188–96. doi: 10.1161/CIRCULATIONAHA.112.122408, PMID: 23319811 PMC3762447

[14] Cassidy A O'Reilly ÉJ Kay C Sampson L Franz M Forman JP. Habitual intake of flavonoid subclasses and incident hypertension in adults. Am J Clin Nutr. (2011) 93:338–47. doi: 10.3945/ajcn.110.006783, PMID: 21106916 PMC3021426

[15] Carvalho MF Lucca ABA Ribeiro ESVR Macedo LR Silva M. Blueberry intervention improves metabolic syndrome risk factors: systematic review and meta-analysis. Nutr Res. (2021) 91:67–80. doi: 10.1016/j.nutres.2021.04.006, PMID: 34139510

[16] Stote KS Burns G Mears K Sweeney M Blanton C. The effect of berry consumption on oxidative stress biomarkers: a systematic review of randomized controlled trials in humans. Antioxidants. (2023) 12:1443. doi: 10.3390/antiox12071443, PMID: 37507981 PMC10376627

[17] McAnulty LS Collier SR Landram MJ Whittaker DS Isaacs SE Klemka JM. Six weeks daily ingestion of whole blueberry powder increases natural killer cell counts and reduces arterial stiffness in sedentary males and females. Nutr Res. (2014) 34:577–84. doi: 10.1016/j.nutres.2014.07.002 25150116

[18] Wood E Hein S Mesnage R Fernandes F Abhayaratne N Xu Y. Wild blueberry (poly)phenols can improve vascular function and cognitive performance in healthy older individuals: a double-blind randomized controlled trial. Am J Clin Nutr. (2023) 117:1306–19. doi: 10.1016/j.ajcnut.2023.03.017 36972800 PMC10315404

[19] Basu A Du M Leyva MJ Sanchez K Betts NM Wu M. Blueberries decrease cardiovascular risk factors in obese men and women with metabolic syndrome. J Nutr. (2010) 140:1582–7. doi: 10.3945/jn.110.124701, PMID: 20660279 PMC2924596

[20] Johnson SA Figueroa A Navaei N Wong A Kalfon R Ormsbee LT. Daily blueberry consumption improves blood pressure and arterial stiffness in postmenopausal women with pre- and stage 1-hypertension: a randomized, double-blind, placebo-controlled clinical trial. J Acad Nutr Diet. (2015) 115:369–77. doi: 10.1016/j.jand.2014.11.001, PMID: 25578927

[21] Curtis PJ van der Velpen V Berends L Jennings A Feelisch M Umpleby AM. Blueberries improve biomarkers of cardiometabolic function in participants with metabolic syndrome-results from a 6-month, double-blind, randomized controlled trial. Am J Clin Nutr. (2019) 109:1535–45. doi: 10.1093/ajcn/nqy380, PMID: 31136659 PMC6537945

[22] Stull AJ Cash KC Champagne CM Gupta AK Boston R Beyl RA. Blueberries improve endothelial function, but not blood pressure, in adults with metabolic syndrome: a randomized, double-blind, placebo-controlled clinical trial. Nutrients. (2015) 7:4107–23. doi: 10.3390/nu7064107, PMID: 26024297 PMC4488775

[23] Woolf EK Terwoord JD Litwin NS Vazquez AR Lee SY Ghanem N. Daily blueberry consumption for 12 weeks improves endothelial function in postmenopausal women with above-normal blood pressure through reductions in oxidative stress: a randomized controlled trial. Food Funct. (2023) 14:2621–41. doi: 10.1039/D3FO00157A, PMID: 36847333

[24] Del Bo C Deon V Campolo J Lanti C Parolini M Porrini M. A serving of blueberry (*V. corymbosum*) acutely improves peripheral arterial dysfunction in young smokers and non-smokers: two randomized, controlled, crossover pilot studies. Food Funct. (2017) 8:4108–17. doi: 10.1039/c7fo00861a, PMID: 29019364

[25] Del Bo C Porrini M Fracassetti D Campolo J Klimis-Zacas D Riso P. A single serving of blueberry (*V. corymbosum*) modulates peripheral arterial dysfunction induced by acute cigarette smoking in young volunteers: a randomized-controlled trial. Food Funct. (2014) 5:3107–16. doi: 10.1039/c4fo00570h, PMID: 25263326

[26] Rodriguez-Mateos A Rendeiro C Bergillos-Meca T Tabatabaee S George TW Heiss C. Intake and time dependence of blueberry flavonoid-induced improvements in vascular function: a randomized, controlled, double-blind, crossover intervention study with mechanistic insights into biological activity. Am J Clin Nutr. (2013) 98:1179–91. doi: 10.3945/ajcn.113.066639 24004888

[27] Bharat D Cavalcanti RRM Petersen C Begaye N Cutler BR Costa MMA. Blueberry metabolites attenuate lipotoxicity-induced endothelial dysfunction. Mol Nutr Food Res. (2018) 62:601. doi: 10.1002/mnfr.201700601, PMID: 29024402 PMC8162272

[28] Rodriguez-Mateos A Istas G Boschek L Feliciano RP Mills CE Boby C. Circulating anthocyanin metabolites mediate vascular benefits of blueberries: insights from randomized controlled trials, metabolomics, and nutrigenomics. J Gerontol A Biol Sci Med Sci. (2019) 74:967–76. doi: 10.1093/gerona/glz047, PMID: 30772905

[29] Halvorsen RE Elvestad M Molin M Aune D. Fruit and vegetable consumption and the risk of type 2 diabetes: a systematic review and dose-response meta-analysis of prospective studies. BMJ Nutr Prev Health. (2021) 4:519–31. doi: 10.1136/bmjnph-2020-000218, PMID: 35028521 PMC8718861

[30] Muraki I Imamura F Manson JE Hu FB Willett WC van Dam RM. Fruit consumption and risk of type 2 diabetes: results from three prospective longitudinal cohort studies. BMJ. (2013) 347:f5001. doi: 10.1136/bmj.f5001, PMID: 23990623 PMC3978819

[31] Stull AJ Cash KC Johnson WD Champagne CM Cefalu WT. Bioactives in blueberries improve insulin sensitivity in obese, insulin-resistant men and women. J Nutr. (2010) 140:1764–8. doi: 10.3945/jn.110.125336, PMID: 20724487 PMC3139238

[32] Stote KS Wilson MM Hallenbeck D Thomas K Rourke JM Sweeney MI. Effect of blueberry consumption on Cardiometabolic health parameters in men with type 2 diabetes: an 8-week, double-blind, randomized, placebo-controlled trial. Curr Dev Nutr. (2020) 4:nzaa030. doi: 10.1093/cdn/nzaa030 32337475 PMC7170047

[33] Krikorian R Skelton MR Summer SS Shidler MD Sullivan PG. Blueberry supplementation in midlife for dementia risk reduction. Nutrients. (2022) 14:1619. doi: 10.3390/nu14081619 35458181 PMC9031005

[34] Curtis PJ Berends L van der Velpen V Jennings A Haag L Chandra P. Blueberry anthocyanin intake attenuates the postprandial cardiometabolic effect of an energy-dense food challenge: results from a double blind, randomized controlled trial in metabolic syndrome participants. Clin Nutr. (2022) 41:165–76. doi: 10.1016/j.clnu.2021.11.030, PMID: 34883305 PMC8757535

[35] Devore EE Kang JH Breteler MM Grodstein F. Dietary intakes of berries and flavonoids in relation to cognitive decline. Ann Neurol. (2012) 72:135–43. doi: 10.1002/ana.23594, PMID: 22535616 PMC3582325

[36] Samieri C Sun Q Townsend MK Rimm EB Grodstein F. Dietary flavonoid intake at midlife and healthy aging in women. Am J Clin Nutr. (2014) 100:1489–97. doi: 10.3945/ajcn.114.085605, PMID: 25411284 PMC4232017

[37] Gao X Cassidy A Schwarzschild MA Rimm EB Ascherio A. Habitual intake of dietary flavonoids and risk of Parkinson disease. Neurology. (2012) 78:1138–45. doi: 10.1212/WNL.0b013e31824f7fc4, PMID: 22491871 PMC3320056

[38] Andres-Lacueva C Shukitt-Hale B Galli RL Jauregui O Lamuela-Raventos RM Joseph JA. Anthocyanins in aged blueberry-fed rats are found centrally and may enhance memory. Nutr Neurosci. (2005) 8:111–20. doi: 10.1080/10284150500078117, PMID: 16053243

[39] Beracochea D Krazem A Henkouss N Haccard G Roller M Fromentin E. Intake of wild blueberry powder improves episodic-like and working memory during Normal aging in mice. Planta Med. (2016) 82:1163–8. doi: 10.1055/s-0042-104419, PMID: 27093246

[40] Casadesus G Shukitt-Hale B Stellwagen HM Zhu X Lee HG Smith MA. Modulation of hippocampal plasticity and cognitive behavior by short-term blueberry supplementation in aged rats. Nutr Neurosci. (2004) 7:309–16. doi: 10.1080/10284150400020482, PMID: 15682927

[41] Rendeiro C Vauzour D Rattray M Waffo-Téguo P Mérillon JM Butler LT. Dietary levels of pure flavonoids improve spatial memory performance and increase hippocampal brain-derived neurotrophic factor. PLoS One. (2013) 8:e63535. doi: 10.1371/journal.pone.0063535, PMID: 23723987 PMC3665790

[42] Shukitt-Hale B Bielinski DF Lau FC Willis LM Carey AN Joseph JA. The beneficial effects of berries on cognition, motor behaviour and neuronal function in ageing. Br J Nutr. (2015) 114:1542–9. doi: 10.1017/S0007114515003451, PMID: 26392037

[43] Poulose SM Bielinski DF Carrihill-Knoll KL Rabin BM Shukitt-Hale B. Protective effects of blueberry- and strawberry diets on neuronal stress following exposure to (56)Fe particles. Brain Res. (2014) 1593:9–18. doi: 10.1016/j.brainres.2014.10.028 25451098

[44] Shukitt-Hale B Carey AN Jenkins D Rabin BM Joseph JA. Beneficial effects of fruit extracts on neuronal function and behavior in a rodent model of accelerated aging. Neurobiol Aging. (2007) 28:1187–94. doi: 10.1016/j.neurobiolaging.2006.05.031, PMID: 16837106

[45] Tan L Yang HP Pang W Lu H Hu YD Li J. Cyanidin-3-O-galactoside and blueberry extracts supplementation improves spatial memory and regulates hippocampal ERK expression in senescence-accelerated mice. Biomed Environ Sci. (2014) 27:186–96. doi: 10.3967/bes2014.007, PMID: 24709099

[46] Carey AN Gomes SM Shukitt-Hale B. Blueberry supplementation improves memory in middle-aged mice fed a high-fat diet. J Agric Food Chem. (2014) 62:3972–8. doi: 10.1021/jf404565s 24446769

[47] Krikorian R Shidler MD Nash TA Kalt W Vinqvist-Tymchuk MR Shukitt-Hale B. Blueberry supplementation improves memory in older adults. J Agric Food Chem. (2010) 58:3996–4000. doi: 10.1021/jf9029332, PMID: 20047325 PMC2850944

[48] McNamara RK Kalt W Shidler MD McDonald J Summer SS Stein AL. Cognitive response to fish oil, blueberry, and combined supplementation in older adults with subjective cognitive impairment. Neurobiol Aging. (2018) 64:147–56. doi: 10.1016/j.neurobiolaging.2017.12.003 29458842 PMC5822748

[49] Miller MG Hamilton DA Joseph JA Shukitt-Hale B. Dietary blueberry improves cognition among older adults in a randomized, double-blind, placebo-controlled trial. Eur J Nutr. (2018) 57:1169–80. doi: 10.1007/s00394-017-1400-8, PMID: 28283823

[50] Krikorian R Kalt W McDonald JE Shidler MD Summer SS Stein AL. Cognitive performance in relation to urinary anthocyanins and their flavonoid-based products following blueberry supplementation in older adults at risk for dementia. J Funct Foods. (2020) 64:103667. doi: 10.1016/j.jff.2019.103667

[51] Cheatham CL Canipe Iii LG Millsap G Stegall JM Chai SC Sheppard KW. Six-month intervention with wild blueberries improved speed of processing in mild cognitive decline: a double-blind, placebo-controlled, randomized clinical trial. Nutr Neurosci. (2023) 26:1019–33. doi: 10.1080/1028415X.2022.2117475, PMID: 36066009

[52] Boespflug EL Eliassen JC Dudley JA Shidler MD Kalt W Summer SS. Enhanced neural activation with blueberry supplementation in mild cognitive impairment. Nutr Neurosci. (2018) 21:297–305. doi: 10.1080/1028415X.2017.1287833, PMID: 28221821 PMC6093614

[53] Bowtell JL Aboo-Bakkar Z Conway ME Adlam AR Fulford J. Enhanced task-related brain activation and resting perfusion in healthy older adults after chronic blueberry supplementation. Appl Physiol Nutr Metab. (2017) 42:773–9. doi: 10.1139/apnm-2016-0550, PMID: 28249119

[54] Cahoon DS Fisher DR Lamon-Fava S Wu D Zheng T Shukitt-Hale B. Blueberry treatment administered before and/or after lipopolysaccharide stimulation attenuates inflammation and oxidative stress in rat microglial cells. Nutr Neurosci. (2023) 26:127–37. doi: 10.1080/1028415X.2021.2020404, PMID: 36692990

[55] Bischoff SC. 'Gut health': a new objective in medicine? BMC Med. (2011) 9:24. doi: 10.1186/1741-7015-9-24, PMID: 21401922 PMC3065426

[56] Staudacher HM Loughman A. Gut health: definitions and determinants. Lancet Gastroenterol Hepatol. (2021) 6:269. doi: 10.1016/S2468-1253(21)00071-6 33714369

[57] Black CJ Drossman DA Talley NJ Ruddy J Ford AC. Functional gastrointestinal disorders: advances in understanding and management. Lancet. (2020) 396:1664–74. doi: 10.1016/S0140-6736(20)32115-2 33049221

[58] Wilder-Smith CH Materna A Olesen SS. Blueberries improve abdominal symptoms, well-being and functioning in patients with functional gastrointestinal disorders. Nutrients. (2023) 15:2396. doi: 10.3390/nu15102396, PMID: 37242279 PMC10223779

[59] Petersen C Bharat D Wankhade UD Kim J-S Cutler BR Denetso C. Dietary blueberry ameliorates vascular complications in diabetic mice possibly through NOX4 and modulates composition and functional diversity of gut microbes. Mol Nutr Food Res. (2022) 66:e2100784. doi: 10.1002/mnfr.202100784 35120277 PMC9132135

[60] Della Lucia CM Oliveira LA Dias KA Pereira SMS da Conceição AR Anandh Babu PV. Scientific evidence for the beneficial effects of dietary blueberries on gut health: a systematic review. Mol Nutr Food Res. (2023) 67:e2300096. doi: 10.1002/mnfr.202300096, PMID: 37428472 PMC10538750

[61] Lee S Keirsey KI Kirkland R Grunewald ZI Fischer JG de La Serre CB. Blueberry supplementation influences the gut microbiota, inflammation, and insulin resistance in high-fat-diet-fed rats. J Nutr. (2018) 148:209–19. doi: 10.1093/jn/nxx027, PMID: 29490092 PMC6251676

[62] Polewski MA Esquivel-Alvarado D Wedde NS Kruger CG Reed JD. Isolation and characterization of blueberry polyphenolic components and their effects on gut barrier dysfunction. J Agric Food Chem. (2020) 68:2940–7. doi: 10.1021/acs.jafc.9b01689, PMID: 31199652

[63] Cladis DP Simpson AMR Cooper KJ Nakatsu CH Ferruzzi MG Weaver CM. Blueberry polyphenols alter gut microbiota & phenolic metabolism in rats. Food Funct. (2021) 12:2442–56. doi: 10.1039/D0FO03457F 33629093 PMC8011555

[64] Lacombe A Li RW Klimis-Zacas D Kristo AS Tadepalli S Krauss E. Lowbush wild blueberries have the potential to modify gut microbiota and xenobiotic metabolism in the rat colon. PLoS One. (2013) 8:e67497. doi: 10.1371/journal.pone.0067497, PMID: 23840722 PMC3696070

[65] Teixeira LD Torrez Lamberti MF DeBose-Scarlett E Bahadiroglu E Garrett TJ Gardner CL. *Lactobacillus johnsonii* N6.2 and blueberry Phytophenols affect Lipidome and gut microbiota composition of rats under high-fat diet. Front Nutr. (2021) 8:757256. doi: 10.3389/fnut.2021.757256, PMID: 34722616 PMC8551501

[66] Pervin M Hasnat MA Lim JH Lee YM Kim EO Um BH. Preventive and therapeutic effects of blueberry (*Vaccinium corymbosum*) extract against DSS-induced ulcerative colitis by regulation of antioxidant and inflammatory mediators. J Nutr Biochem. (2016) 28:103–13. doi: 10.1016/j.jnutbio.2015.10.006, PMID: 26878787

[67] Ntemiri A Ghosh TS Gheller ME Tran TTT Blum JE Pellanda P. Whole blueberry and isolated polyphenol-rich fractions modulate specific gut microbes in an in vitro Colon model and in a pilot study in human consumers. Nutrients. (2020) 12:2800. doi: 10.3390/nu12092800, PMID: 32932733 PMC7551244

[68] Guglielmetti S Fracassetti D Taverniti V Del Bo C Vendrame S Klimis-Zacas D. Differential modulation of human intestinal bifidobacterium populations after consumption of a wild blueberry (*Vaccinium angustifolium*) drink. J Agric Food Chem. (2013) 61:8134–40. doi: 10.1021/jf402495k, PMID: 23883473

[69] Jiao X Wang Y Lin Y Lang Y Li E Zhang X. Blueberry polyphenols extract as a potential prebiotic with anti-obesity effects on C57BL/6 J mice by modulating the gut microbiota. J Nutr Biochem. (2019) 64:88–100. doi: 10.1016/j.jnutbio.2018.07.008, PMID: 30471564

[70] Liu J Hao W He Z Kwek E Zhu H Ma N. Blueberry and cranberry anthocyanin extracts reduce bodyweight and modulate gut microbiota in C57BL/6 J mice fed with a high-fat diet. Eur J Nutr. (2021) 60:2735–46. doi: 10.1007/s00394-020-02446-3, PMID: 33392758

[71] Morissette A Kropp C Songpadith JP Junges Moreira R Costa J Mariné-Casadó R. Blueberry proanthocyanidins and anthocyanins improve metabolic health through a gut microbiota-dependent mechanism in diet-induced obese mice. Am J Physiol Endocrinol Metab. (2020) 318:E965–80. doi: 10.1152/ajpendo.00560.2019, PMID: 32228321

[72] Si X Bi J Chen Q Cui H Bao Y Tian J. Effect of blueberry anthocyanin-rich extracts on peripheral and hippocampal antioxidant defensiveness: the analysis of the serum fatty acid species and gut microbiota profile. J Agric Food Chem. (2021) 69:3658–66. doi: 10.1021/acs.jafc.0c07637, PMID: 33709697

[73] Yan Z Yang F Hong Z Wang S Jinjuan Z Han B. Blueberry attenuates liver fibrosis, protects intestinal epithelial barrier, and maintains gut microbiota homeostasis. Can J Gastroenterol Hepatol. (2019) 2019:1–11. doi: 10.1155/2019/5236149 PMC6893245 31886154

[74] Rodríguez-Daza MC Daoust L Boutkrabt L Pilon G Varin T Dudonné S. Wild blueberry proanthocyanidins shape distinct gut microbiota profile and influence glucose homeostasis and intestinal phenotypes in high-fat high-sucrose fed mice. Sci Rep. (2020) 10:2217. doi: 10.1038/s41598-020-58863-1, PMID: 32041991 PMC7010699

[75] Carey CC Lucey A Doyle L. Flavonoid containing polyphenol consumption and recovery from exercise-induced muscle damage: a systematic review and Meta-analysis. Sports Med. (2021) 51:1293–316. doi: 10.1007/s40279-021-01440-x 33687663

[76] Gonçalves AC Nunes AR Falcão A Alves G Silva LR. Dietary effects of anthocyanins in human health: a comprehensive review. Pharmaceuticals. (2021) 14:690. doi: 10.3390/ph14070690, PMID: 34358116 PMC8308553

[77] Kimble R Jones K Howatson G. The effect of dietary anthocyanins on biochemical, physiological, and subjective exercise recovery: a systematic review and meta-analysis. Crit Rev Food Sci Nutr. (2023) 63:1262–76. doi: 10.1080/10408398.2021.1963208, PMID: 34402657

[78] Rickards L Lynn A Harrop D Barker ME Russell M Ranchordas MK. Effect of polyphenol-rich foods, juices, and concentrates on recovery from exercise induced muscle damage: a systematic review and Meta-analysis. Nutrients. (2021) 13:2988. doi: 10.3390/nu13092988, PMID: 34578866 PMC8465563

[79] Ruiz-Iglesias P Gorgori-González A Massot-Cladera M Castell M Pérez-Cano FJ. Does flavonoid consumption improve exercise performance? Is it related to changes in the immune system and inflammatory biomarkers? A systematic review of clinical studies since 2005. Nutrients. (2021) 13:1132. doi: 10.3390/nu13041132, PMID: 33808153 PMC8065858

[80] Avendano EE Raman G. Blueberry consumption and exercise: gap analysis using evidence mapping. J Altern Complement Med. (2021) 27:3–11. doi: 10.1089/acm.2020.0236, PMID: 33058743

[81] Cook MD Willems MET. Dietary anthocyanins: a review of the exercise performance effects and related physiological responses. Int J Sport Nutr Exerc Metab. (2019) 29:322–30. doi: 10.1123/ijsnem.2018-0088, PMID: 30160565

[82] Souza TCM Goston JL Martins-Costa HC Minighin EC Anastácio LR. Can anthocyanins reduce delayed onset muscle soreness or are we barking up the wrong tree? Prev Nutr Food Sci. (2022) 27:265–75. doi: 10.3746/pnf.2022.27.3.265, PMID: 36313058 PMC9585400

[83] Park CH Kwak YS Seo HK Kim HY. Assessing the values of blueberries intake on exercise performance, TAS, and inflammatory factors. Iran J Public Health. (2018) 47:27–32. PMID: 30186809 PMC6124147

[84] McAnulty LS Nieman DC Dumke CL Shooter LA Henson DA Utter AC. Effect of blueberry ingestion on natural killer cell counts, oxidative stress, and inflammation prior to and after 2.5 h of running. Appl Physiol Nutr Metab. (2011) 36:976–84. doi: 10.1139/h11-120 22111516

[85] McAnulty SR McAnulty LS Nieman DC Dumke CL Morrow JD Utter AC. Consumption of blueberry polyphenols reduces exercise-induced oxidative stress compared to vitamin C. Nutr Res. (2004) 24:209–21. doi: 10.1016/j.nutres.2003.10.003

[86] McLeay Y Barnes MJ Mundel T Hurst SM Hurst RD Stannard SR. Effect of New Zealand blueberry consumption on recovery from eccentric exercise-induced muscle damage. J Int Soc Sports Nutr. (2012) 9:19. doi: 10.1186/1550-2783-9-19, PMID: 22564864 PMC3583121

[87] Nieman DC Gillitt ND Chen GY Zhang Q Sha W Kay CD. Blueberry and/or Banana consumption mitigate arachidonic, cytochrome P450 Oxylipin generation during recovery from 75-km cycling: a randomized trial. Front Nutr. (2020) 7:121. doi: 10.3389/fnut.2020.00121, PMID: 32850939 PMC7426440

[88] Nieman DC Sakaguchi CA Omar AM Davis KL Shaffner CE Strauch RC. Blueberry intake elevates post-exercise anti-inflammatory oxylipins: a randomized trial. Sci Rep. (2023) 13:11976. doi: 10.1038/s41598-023-39269-1, PMID: 37488250 PMC10366094

[89] Bloedon T Vendrame S Bolton J Lehnhard R Riso P Klimis-Zacas D. The effect of wild blueberry (*Vaccinium angustifolium*) consumption on oxidative stress, inflammation, and DNA damage associated with exercise. Comp Exer Physiol. (2015) 11:173–81. doi: 10.3920/CEP150014

[90] U.S. Department of Agriculture & U.S. Department of Health and Human Services. Dietary guidelines for Americans, 2020–2025. 9th ed (2020).

[91] Pérez-Jiménez J Neveu V Vos F Scalbert A. Identification of the 100 richest dietary sources of polyphenols: an application of the phenol-explorer database. Eur J Clin Nutr. (2010) 64:S112–20. doi: 10.1038/ejcn.2010.221, PMID: 21045839

[92] Blomhoff R Andersen R Arnesen EK Christensen JJ Eneroth H Erkkola M. Nordic nutrition recommendations 2023. Copenhagen: Nordic Council of Ministers (2023).

[93] Li L Pegg RB Eitenmiller RR Chun J-Y Kerrihard AL. Selected nutrient analyses of fresh, fresh-stored, and frozen fruits and vegetables. J Food Compos Anal. (2017) 59:8–17. doi: 10.1016/j.jfca.2017.02.002

[94] Muñoz-Fariña O López-Casanova V García-Figueroa O Roman-Benn A Ah-Hen K Bastias-Montes JM. Bioaccessibility of phenolic compounds in fresh and dehydrated blueberries (*Vaccinium corymbosum* L.). Food Chem Adv. (2023) 2:100171. doi: 10.1016/j.focha.2022.100171

